# Venovenous Extracorporeal Membrane Oxygenation for Acute Respiratory Distress Syndrome in Adults: Prognostic Factors for Outcomes

**DOI:** 10.1097/MD.0000000000002870

**Published:** 2016-03-03

**Authors:** Meng-Yu Wu, Chung-Chi Huang, Tzu-I Wu, Chin-Liang Wang, Pyng-Jing Lin

**Affiliations:** From the Department of Cardiovascular Surgery (M-YW, P-JL); Department of Thoracic Medicine, Chang Gung Memorial Hospital and Chang Gung University (C-CH, C-LW) and Department of Obstetrics and Gynecology, Wan Fang Hospital, Taipei Medical University (T-IW), Taipei, Taiwan.

## Abstract

Despite a therapeutic option for severe acute respiratory distress syndrome (ARDS), the survival benefit of venovenous extracorporeal membrane oxygenation (VV-ECMO) is still controversial in adults. This study was aimed at investigating the prognostic factors for ECMO-treated ARDS in adult patients.

From 2012 to 2015, 49 patients (median age: 57 years) received VV-ECMO in our institution and were included in this retrospective study. The indication of VV-ECMO was a severe hypoxemia (P_a_O_2_/FiO_2_ ratio <70 mmHg) under mechanical ventilation (MV) with a peak inspiratory pressure (PIP) >35 cmH_2_O and a F_i_O_2_ >0.8. To decrease the impact of pulmonary injuries associated with the high-pressure ventilation, the settings of MV on VV-ECMO were downgraded according to our protocol. Outcomes of this study were death on VV-ECMO and death in hospital. Important demographic and clinical data during the treatment were collected for outcome analyses.

All patients experienced significant improvements in arterial oxygenation on VV-ECMO. Twenty-four hours after initiation of VV-ECMO, the median P_a_O_2_/FiO_2_ ratio increased from 58 to 172 mmHg (*P* < 0.001) and the median S_a_O_2_ increased from 86% to 97% (*P* < 0.001). In the meantime, the MV settings were also effectively downgraded. The median PIP decreased from 35 to 29 cmH_2_O (*P* < 0.001) and the median tidal volume decreased from 7 to 5 ml/kg/min (*P* < 0.001). Twelve patients died during the treatment of VV-ECMO and 21 patients died before hospital discharge. Among all of the pre-ECMO variables, the pre-ECMO pulmonary dynamic compliance (PC_dyn_) <20 mL/cmH_2_O was identified to be the prognostic factor of death on VV-ECMO (odds ratio [OR]: 6, 95% confidence interval [CI]: 1–35, *P* = 0.03), and the pre-ECMO duration of MV >90 hours was the prognostic factor of death before hospital discharge (OR: 7, 95% CI: 1–29, *P* = 0.01).

VV-ECMO was a useful salvage therapy for severe ARDS in adults. However, the value of PC_dyn_ and the duration of MV before intervention with VV-ECMO may significantly affect the patients’ outcomes.

## INTRODUCTION

Acute respiratory failure is both the most common and lethal single organ failure in intensive care units.^1^ Severe acute respiratory distress syndrome (ARDS) is the most serious form of this condition and carries a mortality rate of 40% to 45%.^2^ The standard treatment of ARDS is divided into 2 parts; the first is to treat the underlying disease, which induces acute lung injury, and the second is to correct arterial hypoxemia resulted from a compromised gas exchange of the injured lungs.^3,4^ Mechanical ventilation (MV) is the primary therapy to correct arterial hypoxemia in this scenario.^3^ However, it may superimpose additional damages on the already injured pulmonary alveoli, since they are very sensitive to the cyclic ventilation delivered by MV.^5,6^ An inadequately high inspiratory pressure may cause overdistension of these alveoli and destroys their infrastructures.^5^ If the alveolar-capillary barrier is destructed, interstitial pulmonary edema may occur and worsen the pulmonary gas exchange.^5,6^ To avoid the above-mentioned ventilator-induced lung injury (VILI), using limited peak inspiratory pressure (PIP) to ventilate the lungs with low tidal volumes (V_T,_ often 4–6 mL/kg/min), is the mainstream strategy of MV for ARDS in adults.^3^ Nevertheless, some patients may still show hypoxemia or hypercapnea under this lung-protective ventilation (LPV).^3,4^ These patients often require an increase of PIP to drive a larger V_T_ for ventilation and therefore have an increased risk of VILI. If the arterial hypoxemia is still unimproved in spite of a high-pressure ventilation, venovenous extracorporeal membrane oxygenation (VV-ECMO) is an option to solve this problem without overstretching the injured lungs.^4,7^ It shunts part of the venous blood from the inferior vena cava to the extracorporeal circuit for gas exchange, and then returns the refreshed blood back to right atrium (RA).^7,8^ Therefore, before entering the native lungs, the blood in pulmonary artery (PA) already carries more oxygen and less CO_2_ than usual. This prepulmonary gas exchange of VV-ECMO should reduce workload on the native lungs, and allows them to be ventilated gently.^7,8^ The reduction of V_T_ also helps the right ventricle (RV) by decreasing the intrathoracic pressure.^9^ Unfortunately, the assumed protective effect of VV-ECMO has not yet brought credible benefits of survival in adult patients with ARDS,^8,10,11^ and the safety of VV-ECMO is also questioned for its traumatizing effect on blood cells.^12,13^ To refine the current inclusion criteria for this potentially helpful but invasive treatment, this study was aimed at investigating the prognostic factors for ARDS in adult patients who were treated with a protocol-guided VV-ECMO.

## MATERIALS AND METHODS

### Study Population

From January 2012 to January 2015, a total of 53 adult patients (age > 18 years) received VV-ECMO for respiratory support at Chang Gung Memorial Hospital. Our indication of VV-ECMO for ARDS was a deteriorating hypoxemia (a P_a_O_2_/FiO_2_ ratio <70 mmHg) under advanced MV. The settings of MV were considered to be advanced when a PIP >35 cmH_2_O and a FiO_2_ >0.8 were required to maintain an arterial oxygen tension ≥60 mmHg. To reduce the risks of unnecessary injury and futile medical acts, VV-ECMO was contraindicated in ARDS patients having an uncontrolled hemorrhage or a known brain damage. Among the 53 patients, 4 patients were excluded from this retrospective study because of death on VV-ECMO in the first 24 hours (n = 3) or being transferred to another institution (n = 1). This study was conducted in accordance with the amended Declaration of Helsinki.^14^ The ethics committee of the Chang Gung Medical Foundation approved the protocol (CGMF IRB no. 104–5178B) and waived the necessity of individual patient consent.

### Data Collection

We collected the results of essential demographic (age, sex) and clinical variables (etiologies, identified pathogens, organ failures and associated scores, daily fluid balance, routine biochemistry testes, MV settings, common indices of arterial oxygenation, duration of VV-ECMO, duration of MV, and hospital days) for each patient. Repeatedly measured variables were collected at the following points if available: before the administration of VV-ECMO, immediately after the administration of VV-ECMO, 24 hours after the administration of VV-ECMO, before the disconnection of VV-ECMO, and 24 hours after the disconnection of VV-ECMO. The primary outcome of this study was death on VV-ECMO and the secondary outcome was death in hospital.

### Protocol of VV-ECMO in Adult ARDS

**FIGURE 1:**
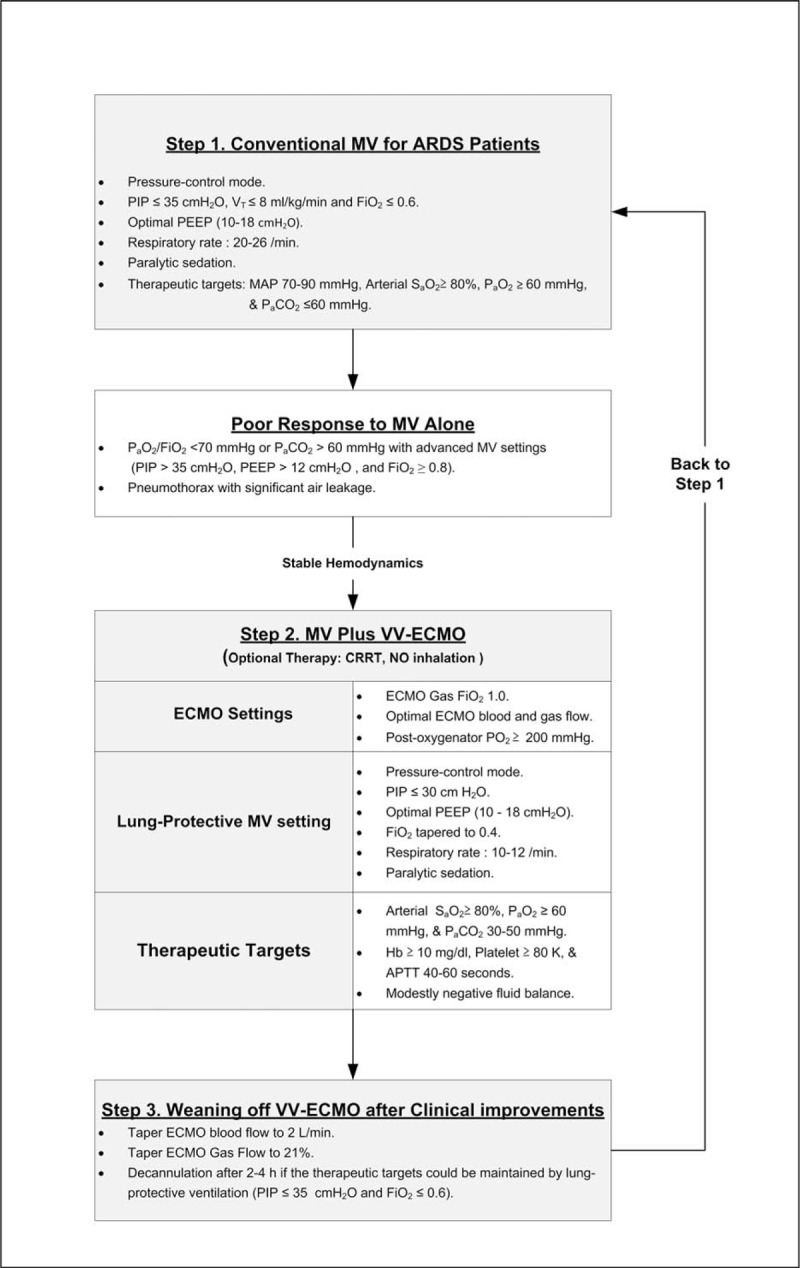
The therapeutic protocol of severe acute respiratory distress syndrome in adults. APTT = activated partial thromboplastin time, ARDS = acute respiratory distress syndrome, F_i_O_2_ = fraction of inspired oxygen, Hb = hemoglobin, MAP = mean arterial pressure, MV = mechanical ventilation, NO = nitric oxide, P_a_O_2_ = arterial oxygen tension, P_a_CO_2_ = arterial tension of carbon dioxide, PEEP = positive end-expiratory pressure, PIP = peak inspiratory pressure, SaO2 = arterial oxygen saturation, V_T_ = tidal volume, VV-ECMO = venovenous extracorporeal membrane oxygenation.

Figure 1 summarizes our therapeutic protocol of VV-ECMO used for ARDS in adults. Our techniques of VV-ECMO are also described in our previous publications.^12,15–17^ We use the Capiox emergent bypass system (Terumo Inc, Tokyo, Japan) and the 2-cannula method (DLP Medtronic, Minneapolis, MN; femoral inflow cannula: 19–23 French, jugular outflow cannula: 17–21 French) to perform VV-ECMO via percutaneous cannulation. Initially, we maximize the sweep gas flow (10 L/min, pure oxygen) to rapidly remove CO_2_, and gradually increase the ECMO pump flow to achieve the best pulse oximetry-detected oxyhemoglobin saturation (S_p_O_2_). To rest the lungs, we downgrade the setting of MV to a lung-protective level step-by-step. At first, we use a pressure-control ventilation (PCV) with a peak airway pressure (PIP) ≤35 cmH_2_O and a moderate positive end expiratory pressure (PEEP; often 12–16 cmH_2_O) to obtain an estimated V_T_ ≤6 mL/kg/min under VV-ECMO. Then we take the arterial and the post-oxygenator blood samples repeatedly to adjust the sweep gas flow and the blood flow of VV-ECMO to provide an optimal arterial oxygenation that allows a downgrade in the PIP (to ≤30 cmH_2_O) and the F_i_O_2_ (to 0.4) of MV. The hemoglobin (Hb) is kept ≥ 10 g/dL to increase the capacity of oxygenation. A modest volume replacement with packed red blood cell is provided to maintain a steady ECMO blood flow ≥3 L/min. We use loop diuretics (usually Furosemide in continuous dripping) or continuous renal replacement therapy (CRRT, in patients with renal shutdown) to achieve a negative fluid balance to dry the lungs.^18,19^ We use systemic heparinization to anticoagulate the patient-ECMO circulation and keep the activated partial thromboplastin time (aPTT) around 40 to 55 seconds. If the patient shows significant improvements, we would try to wean him/her from VV-ECMO as long as the arterial oxygenation could be maintained under the LPV (PIP ≤35cmH_2_O and MV F_i_O_2_ ≤0.6).

### Statistical Analysis

Statistical analyses were performed with SPSS for Windows (Version 15.0, SPSS, Inc, IL). For all analyses, the statistical significance was set at *P* < 0.05. Because the dataset was relative small, nonparametric methods including the Mann–Whitney *U* test, the Kruskal-Wallis test, and the Wilcoxon signed ranks tests were used for univariate comparisons of the independent and the paired numerical variables. The Pearson or Spearman correlation test was used to test the linear relationship between numerical variables. The *χ*^2^ or Fisher exact test was used to compare the categorical variables. Data were presented as median (interquartile range; IQR) for the numerical variables and as percentage for the categorical variables. The multivariate logistic regression method was used to identify independent predictors of death before weaning off VV-ECMO or death before hospital discharge. For practical use, numerical variables showing a *p* < 0.05 with the simple logistic regression were dichotomized by the cutoff values that were determined by the receiver-operating characteristic (ROC) curve analysis. The point that showed the best Youden index (sensitivity + specificity −1) was chosen to be the cutoff value.^20^ The newly dichotomized variables were retested with the multivariate logistic regression method to create a new prediction model. The prediction model was also tested by the Hosmer-Lemeshow test for its goodness-of-fit.

## RESULTS

### Demographics, Improvements of Arterial Oxygenation, and the Outcomes

The causes of ARDS included pneumonia (n = 30; bacterial 11, viral 8, fungal 1, and unidentified pathogen 10), trauma (n = 9), intra-abdominal infection (n = 5), and others (n = 5; 1 hypoxemia after pulmonary endarterectomy and 4 hypoxemia in fluid-overloaded patients with acute-on-chronic renal diseases). The MV modes before VV-ECMO were PCV (n = 48) and volume-controlled ventilation (n = 1). Seventeen patients experienced airway pressure release ventilation and 1 patient had nitric oxide inhalation before VV-ECMO. None of them was exposed to high frequency oscillatory ventilation or prone position, and all of them were paralyzed with neuromuscular agents before VV-ECMO. Twenty-eight patients achieved the goals of LPV during VV-ECMO (Figure 1). Table 1 demonstrates the comparisons of arterial blood gas (ABG) and MV data before and after 24 hours of intervention with VV-ECMO. The median P_a_O_2_/FiO_2_ ratio increased from 58 to 172 mmHg (*P* < 0.001) and the median S_a_O_2_ increased from 86% to 97% (*P* < 0.001) after 24 hours of intervention with VV-ECMO. In the meantime, the MV settings were also downgraded. The median PIP decreased from 35 to 29 cmH_2_O (*P* < 0.001) and the median tidal volume decreased from 7 to 5 mL/kg/min (*P* < 0.001). During VV-ECMO, 3 patients experienced pneumothorax and 12 patients experienced major hemorrhages that required endoscopic or surgical interventions. The sources of hemorrhage were gastrointestinal tract (n = 5), cerebral (n = 3), airway (n = 1), urinary bladder (n = 1), hemothorax (n = 1; a complication of pneumothorax drainage), and retroperitoneum (n = 1, in a trauma patient). Twelve patients died during the treatment of VV-ECMO and 21 patients died before hospital discharge. The causes of death in the 21 patients were sepsis and multiple organ failure syndrome (n = 15), cerebral damages (n = 3; 2 cerebral hemorrhages and 1 hypoxic encephalopathy), uncontrolled hemorrhages (n = 2; 1 hemothorax and 1 retroperitoneal hemorrhage), cardiac arrest owing to unknown reason (n = 1). Thirty-two patients developed severe renal failure and required CRRT during VV-ECMO (n = 30) or after its removal (n = 2).

**TABLE 1:**
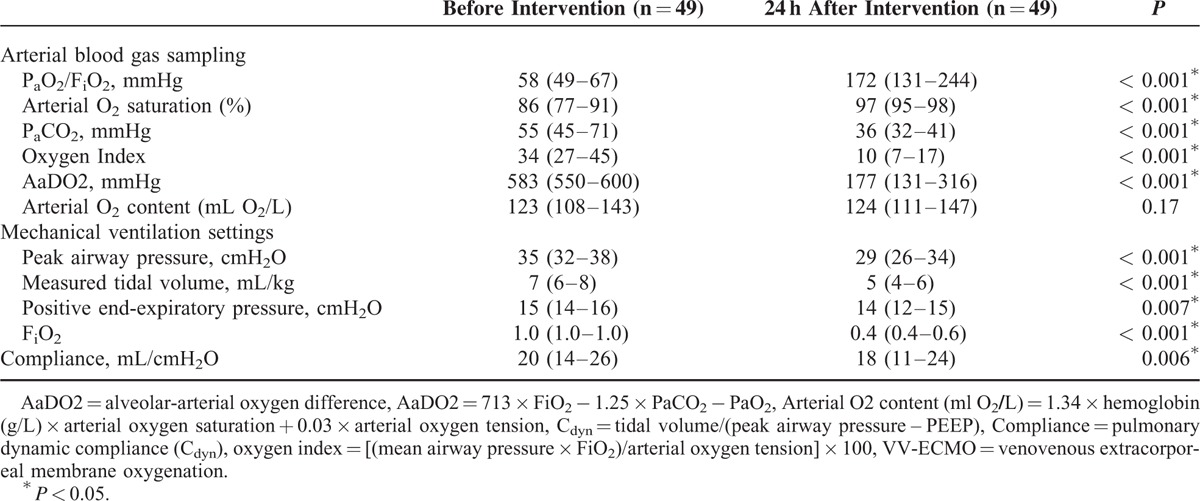
Comparisons of Data in Arterial Blood Gas Sampling and the Settings of Mechanical Ventilation Before and After 24 hours of Intervention With VV-ECMO

### Univariate and Multivariate Analyses of Outcomes

**FIGURE 2:**
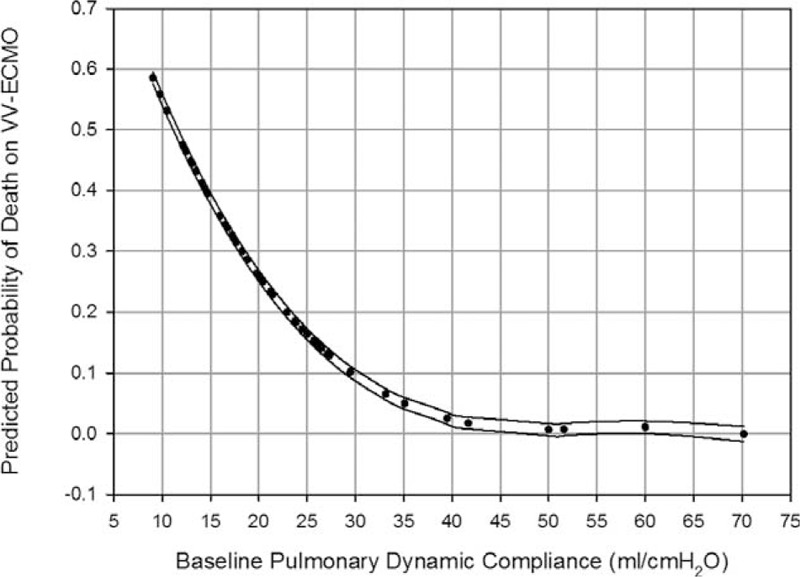
Relationship between the estimated probability of death on venovenous extracorporeal membrane oxygenation (VV-ECMO) and the baseline pulmonary dynamic compliance (PC_dyn_). The curves that present the 95% confidence interval corresponding to the observed PC_dyn_ were also plotted. (Baseline PC_dyn_: the PC_dyn_ just before the intervention with VV-ECMO.).

**FIGURE 3:**
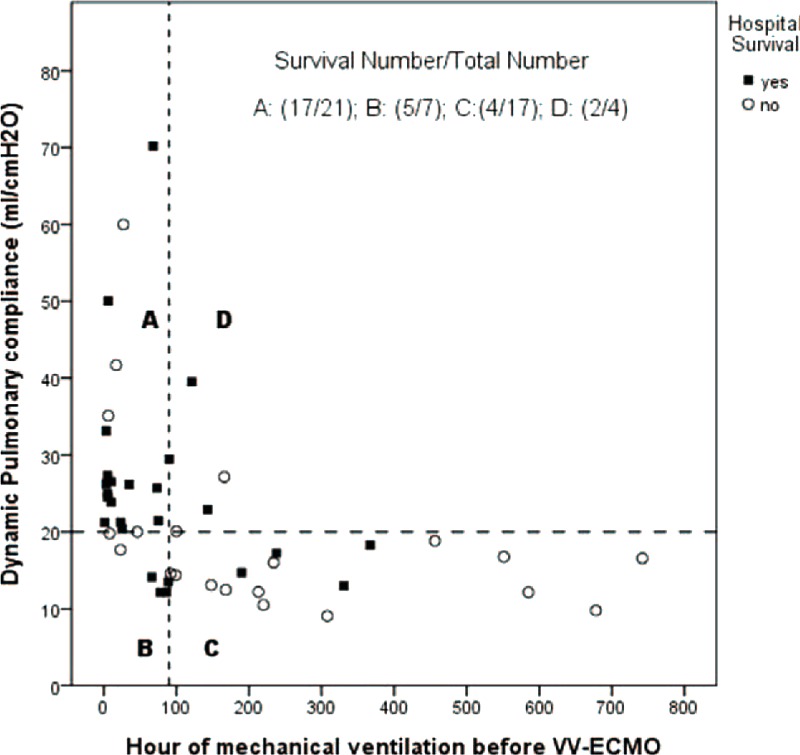
The scatter plot of the pulmonary dynamic compliance and the duration of mechanical ventilation before the intervention with venovenous extracorporeal membrane oxygenation.

Table 2 and Table 3 demonstrate the results of univariate analyses for the outcomes. According to the 2 tables, the data of pre-ECMO pulmonary dynamic compliance (PC_dyn_), pre-ECMO duration of MV, incidence of major hemorrhage on VV-ECMO, and incidence of renal dialysis during the treatment had significantly different distributions among patients with different outcomes. After being tested with ROC curve analysis and multivariate logistic regression, pre-ECMO PC_dyn_ <20 mL/cmH_2_O was identified to be the only independent predictor of death on VV-ECMO (odds ratio [OR]: 6, 95% confidence interval [CI]: 1–35, *P* = 0.03). This predictive model fitted the dataset well (Hosmer-Lemeshow test: *χ*^2^ = 1.2, *P* = 0.54) and had a good predictive power (c-index: 0.75). The sensitivity, specificity, positive predictive value (PPV), and negative predictive value (NPV) of this cutoff value of pre-ECMO PC_dyn_ were 83%, 62%, 42%, and 92%. Figure 2 demonstrates the relationship between the estimated risk of death-on-ECMO and the value of pre-ECMO PC_dyn_. The same procedures were used to find the independent predictors of death before hospital discharge. Pre-ECMO duration of MV >90 hours (OR: 7, 95% CI: 1–29, *P* = 0.01) and major hemorrhages on VV-ECMO (OR: 21, 95% CI: 2–209, *P* = 0.008) were identified to be the independent predictors of death before hospital discharge. This predictive model also fitted the dataset well (Hosmer-Lemeshow test: *χ*^2^ = 0.7, *P* = 0.7) and had a good predictive power (c-index: 0.84). The sensitivity, specificity, PPV, and NPV of this cutoff value of pre-ECMO duration of MV were 71%, 79%, 71%, and 79%. Figure 3 illustrates a scatter plot of the 2 independent predictors in our cohort.

**TABLE 2:**
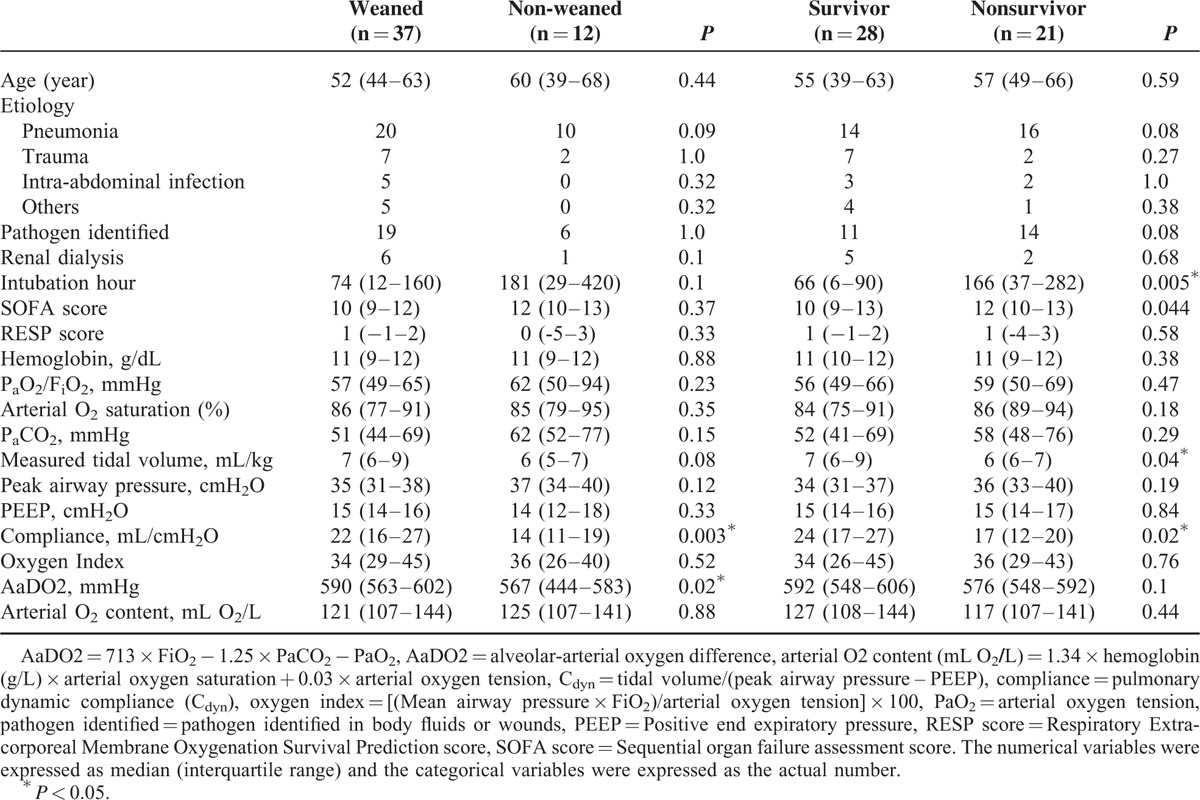
Demographic and Clinical Parameters Before Intervention With Venovenous Extracorporeal Membrane Oxygenation

**TABLE 3:**
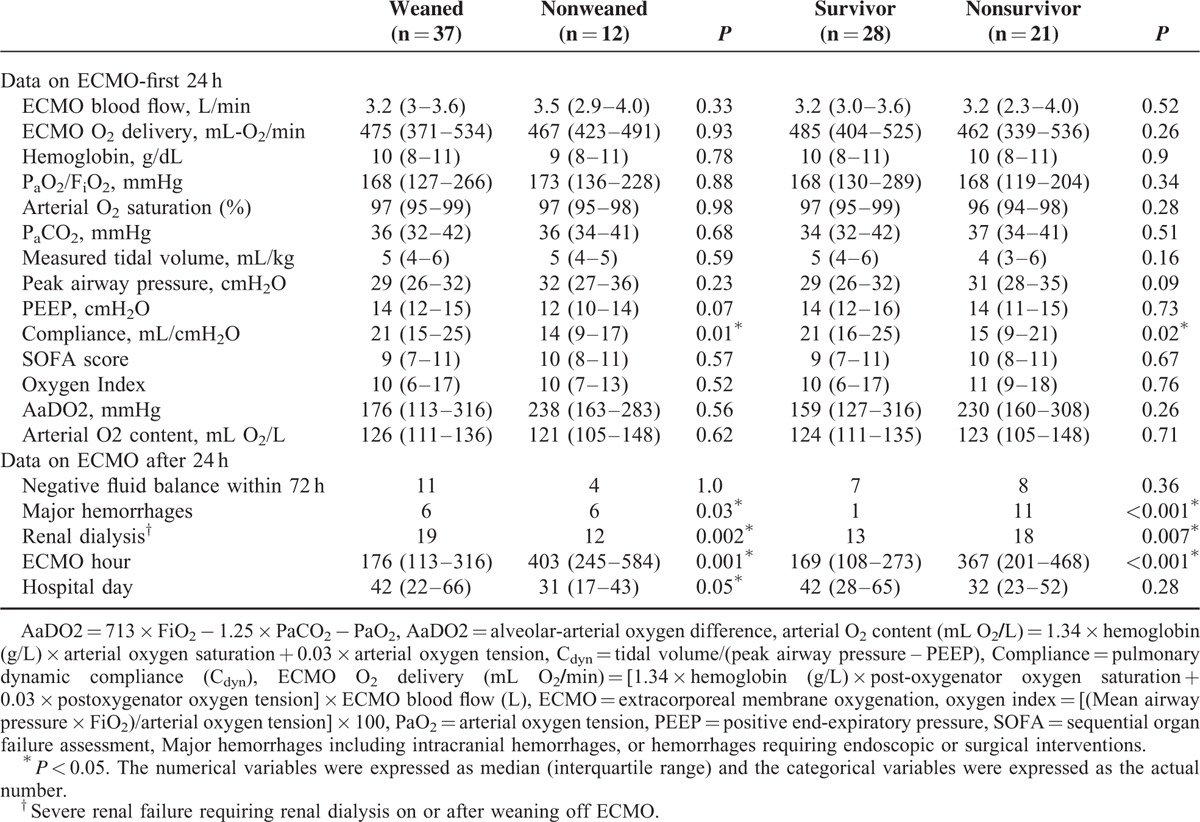
Laboratory and Clinical Data Collected During and After Removal of Venovenous Extracorporeal Membrane Oxygenation

## DISCUSSION

This study was aimed at identifying the prognostic factors for ARDS in adult patients who were treated with VV-ECMO. VV-ECMO has become an attractive option of advanced respiratory support for adults since the 2009 H_1_N_1_ influenza pandemics.^21^ A great deal of research has been directed toward measuring the survival benefits of VV-ECMO.^10,22,23^ Despite not providing definite answers, these studies have indicated that ECMO expands the armamentarium for treating ARDS in adult patients. Developing a therapeutic protocol based on institutional expertise and available resources is necessary to handle this complex therapy.^8^ From a historic review of our ARDS patients treated during 2007 to 2012,^17^ we found that the ECMO patients had a superior hospital survival rate when compared with patients treated with MV alone, if the age and disease severity were matched (survival rate: 51% vs 24%, *P* = 0.009). So we now put VV-ECMO as the respiratory support next to conventional MV in our management of ARDS in adults (Figure 1). In the current study, about half of the supported patients responded to VV-ECMO and recovered from severe ARDS. In the other half of the patients, for unknown reasons, VV-ECMO just postponed but not prevented their death from the progression of the original diseases, although a temporary improvement in arterial oxygenation was seen. Even worse, this salvage therapy caused fatal hemorrhages in some of these patients. According to the Extracorporeal Life Support Organization (ELSO) Registry Report 2012,^24^ the incidence of lethal intracranial hemorrhages is 3.2% in ECMO used for adult respiratory failure. The risk of ECMO-associated hemorrhage is difficult to be eliminated and it is elevated when the stay on ECMO is increased.^25^ Thus, identifying the prognostic factors among the pre-ECMO variables may provide important clues in redefining the current intervention criteria for VV-ECMO to achieve a successful rescue operation.

Among the pre-ECMO variables included in the present study, the duration of MV and PC_dyn_ was revealed to be the prognostic factor of outcomes. A negative correlation was also found between these 2 factors (*r* = −0.4, *P* = 0.006). Pre-ECMO duration of MV is a well-known predictor of hospital mortality in ECMO-treated patients and has been included in the outcome prediction models (RESP score^26^ and SAVE score^27^) developed by ELSO from its registry database. Pre-ECMO duration of MV >7 days is a common exclusion criterion in prospective studies focusing on survival benefits of VV-ECMO in adults with ARDS.^10,28^ The reason why the increase of pre-ECMO duration of MV has negative impacts on the outcomes of ECMO-treated patients is uncertain, but VILI should play an important role in this process. According to previous studies of VILI, alveolar overdistension is the pathogenesis of VILI and often induces a reduction of pulmonary compliance (PC).^5,6^ Thus, we may take the reduced PC as a rough index of the severity of VILI before considering intervention with VV-ECMO. We choose the PC_dyn_ as the target PC here because the pulmonary dynamic strain is considered to be more important than the static strain in the pathogenesis of VILI.^29^ The median value of pre-ECMO PC_dyn_ in our cohort was 20 mL/cmH_2_O, which is a value <10% of the normal PC_dyn_.^30^ According to Figure 2, the risk of death-on-ECMO was >25% in patients that had a PC_dyn_ <20 mL/cmH_2_O before intervention with VV-ECMO. This risk may be minimized in patients having a PC_dyn_ ≥40 mL/cmH_2_O before intervention. To provide a comprehensive view on the 2 correlated prognostic factors, a scatter plot was made to observe their distributions among patients to evaluate their synergistic effect on hospital mortality. According to Figure 3, the patient group with a combination of PC_dyn_ <20 cmH_2_O and duration of MV >90 hours before intervention had a significantly lower hospital survival rate than the group with a combination of PC_dyn_ ≥20 cmH_2_O and duration of MV ≥90 hours (hospital survival rate: 24% vs 81%, *P* < 0.001). Table 4 shows the relationship between the deterioration of ventilatory parameters and the duration of pre-ECMO MV. Compared with patients with a pre-ECMO MV period ≤7 days, the patients with a pre-ECMO MV period >7 days did present a less severe hypoxemia in the beginning of MV. However, they experienced a significant loss of PC_dyn_ on MV while the conventional threshold of hypoxemia for VV-ECMO was eventually reached, and they finally exhibited an inferior survival to the other 2 groups (31% vs 67%, *P* = 0.05). It is surprising that the group with the best arterial oxygenation at the beginning of MV had the worst chance of survival on VV-ECMO. This finding reflected a truth: we might have a delay in administering VV-ECMO to some patients in need, especially to patients showing a moderate but deteriorating hypoxemia. There are 2 potential ways to solve this problem, to reduce the threshold of hypoxemia for VV-ECMO or to expand the inclusion criteria of VV-ECMO. In this study, the PF ratios and the values of PC_dyn_ (both were obtained in a given time point) were independent of each other. Therefore, we considered that both the duration of MV and the PC_dyn_ were practical pre-interventional references to the hospital mortality of VV-ECMO. Surveillance of PC_dyn_ in adult patients with moderate ARDS may be helpful to the early detection of proper candidates for VV-ECMO. Based on our results, we suggested that the critical value of PC_dyn_ should be 20 mL/cmH_2_O. This is consistent with the result of a recently published international multicenter retrospective study of VV-ECMO for adult respiratory failure.^31^ In that study, the median pulmonary compliance in the survivors is superior to the value in the nonsurvivors (23.3 vs 16.7 mL/cmH_2_O; *p* = 0.003). Nevertheless, more studies are needed to define the optimal PC_dyn_, which can accurately predict the outcomes of this salvage therapy.

**TABLE 4:**
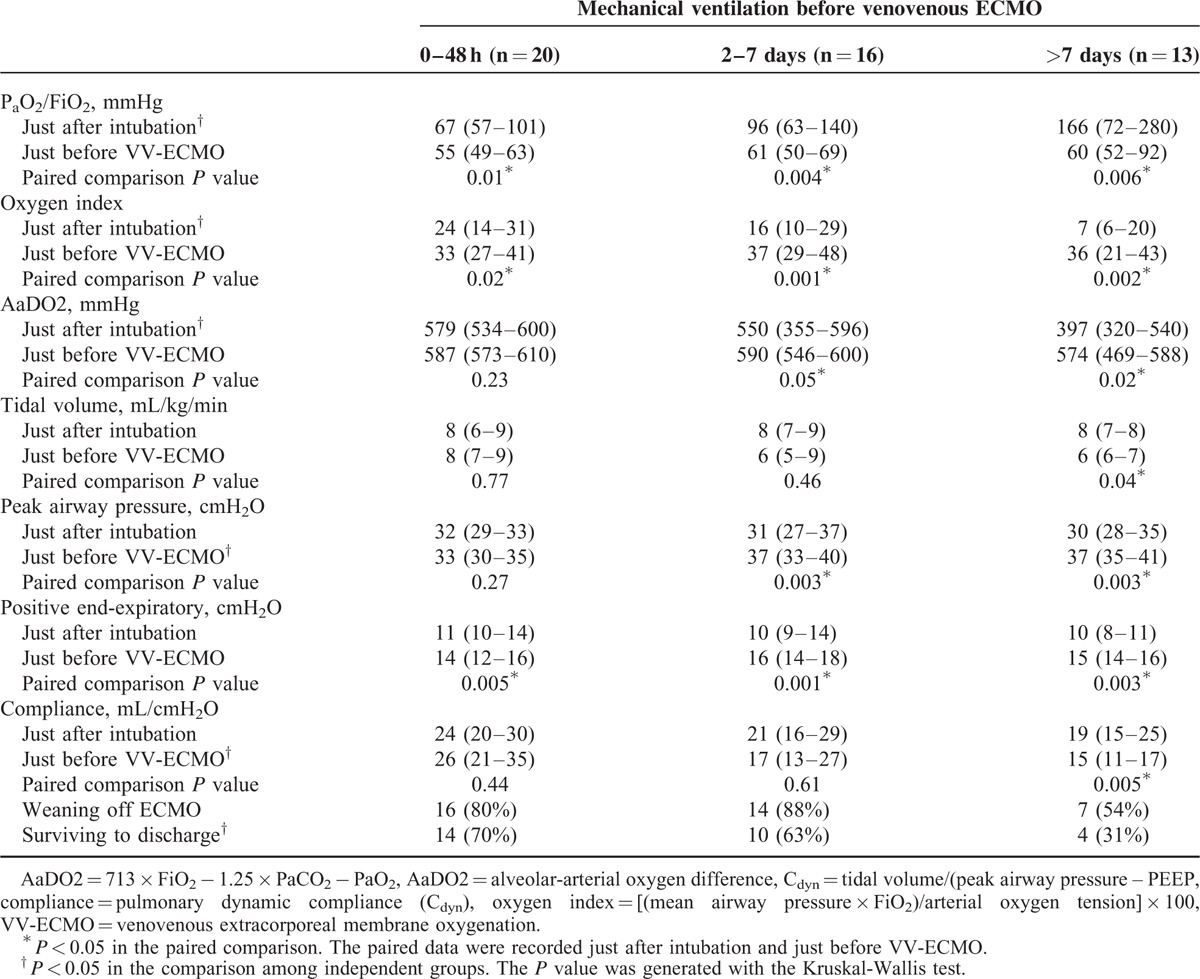
Changes of Ventilatory Parameters Since the Beginning of Mechanical Ventilation to the Administration of VV-ECMO

Finally, we wanted to define the pulmonary recovery associated with VV-ECMO. Table 5 shows changes in the ABG and MV data before the beginning of VV-ECMO and after its disconnection in the survivors. The efficiency of blood gas exchange was the most prominent recovery in these survivors. The PC_dyn_ had only an insignificant improvement to the pre-ECMO level. This result may re-emphasize the importance of a preserved PC_dyn_ to the success of VV-ECMO. In patients with a preserved potential of pulmonary recruitment, the recruitable alveoli may participate in gas exchange again when they are ventilated adequately on VV-ECMO. This process should improve the regional ventilation-perfusion mismatch and reduce the intrapulmonary shunting. With the sustained work of recritable alveoli, the patient may finally achieve an acceptable gas exchange and be weaned off VV-ECMO. Again, further studies are necessary to support this viewpoint and to validate the lowest limit of PC_dyn_ required for a successful rescue with VV-ECMO.

**TABLE 5:**
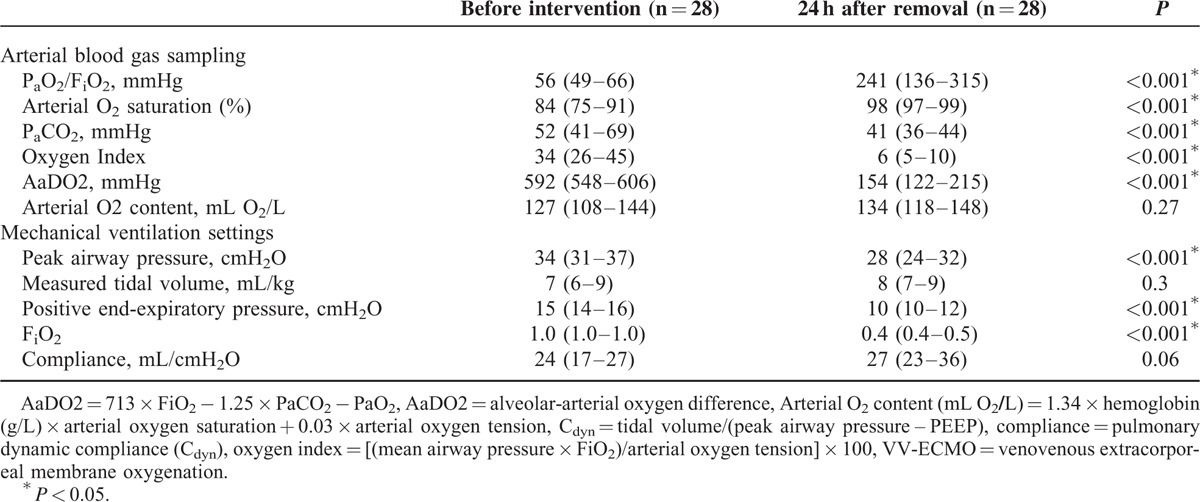
Comparisons of Data in Arterial Blood Gas Sampling and the Settings of Mechanical Ventilation Before Intervention With VV-ECMO and After 24 hours of its Removal in the Survivors

The limitations of this study are its retrospective design and relative small number of cases involved. A full assessment of the therapeutic impacts of ECMO on adults with ARDS was not achieved because only the patients treated with VV-ECMO were included. Further prospective and collaborative studies involving a large population and an integrated protocol in data acquisition and interpretation are necessary to optimize the analysis of the therapeutic effects of ECMO in adults with ARDS.

## CONCLUSION

VV-ECMO was a useful salvage therapy for severe ARDS in adults. It provided physicians a chance to break the vicious cycle of VILI and hypoxemia in these extremely ill patients. However, the value of PC_dyn_ and the duration of MV before intervention with VV-ECMO may significantly affect the patients’ outcomes.
